# Physicochemical Properties and Transdermal Absorption of a Flurbiprofen and Lidocaine Complex in the Non-Crystalline Form

**DOI:** 10.3390/pharmaceutics15020318

**Published:** 2023-01-18

**Authors:** Qihui Xu, Takayuki Furuishi, Kaori Fukuzawa, Etsuo Yonemochi

**Affiliations:** 1 Department of Physical Chemistry, School of Pharmacy and Pharmaceutical Sciences, Hoshi University, 2-4-41 Ebara, Shinagawa-ku, Tokyo 142-8501, Japan; 2 Graduate School of Pharmaceutical Sciences, Osaka University, 1-6 Yamadaoka, Suita 565-0871, Japan

**Keywords:** flurbiprofen, lidocaine, molecular complex, amorphous, transdermal

## Abstract

Amorphous drug formulations exploiting drug–drug interactions have been extensively studied. This study aims to develop a transdermal system containing an amorphous complex of the nonsteroidal anti-inflammatory drug (NSAID) flurbiprofen (FLU) and lidocaine (LDC) for alleviating chronic pain. The high-viscosity complex between FLU and LDC (Complex) was obtained by heating in ethanol. For the complex, attenuated total reflection-Fourier transform infrared spectroscopy showed a shift in the carboxy-group-derived peak of FLU, and differential scanning calorimetry indicated the endothermic peaks associated with the melting of FLU and LDC disappeared. ^13^C dipolar decoupling and ^15^N cross-polarization magic-angle spinning nuclear magnetic resonance measurement suggested the interaction between the carboxyl group of FLU and the secondary amine of LDC. The interaction between the aromatic rings of FLU and LDC contributed to the molecular complex formation. The solubility of FLU from the complex was about 100 times greater than FLU alone. The skin permeation flux of FLU from the complex through the hairless mouse skin was 3.8 times higher than FLU alone in hypromellose gel. Thus, adding LDC to the formulation can be an effective method for enhancing the skin permeation of NSAIDs, which can prove useful for treating chronic pain and inflammatory diseases.

## 1. Introduction

For the past half-century, researchers have been studying molecular complexes to improve the physicochemical characteristics of drugs. Recently, amorphous drug formulations that exploit drug–drug interactions have been intensively studied [1]. Drugs in the amorphous state are generally in a higher energy state, making them more soluble in water than crystals. However, it has poor stability and usually transfers to crystals with a low energy state. Therefore, it is difficult to keep the drug substance stable in an amorphous state for a long time. In recent years, the amorphization of drugs by molecular complexing represented by co-amorphous [2], ionic liquids (ILs) [3], and deep eutectic solvents (DESs) [4] has attracted attention for improving the physical properties of drugs and stabilizing them.

A co-amorphous system is a multi-component, single-phase amorphous solid system linked by weak intermolecular interactions between the components, such as hydrogen bonds of carboxylic acids, phenols/alcohols, and carboxamides [5]. ILs are formed from a combination of organic heterocyclic cations and organic or inorganic anions and are liquids at temperatures <100 °C [6]. In contrast, DESs are systems formed by eutectic mixtures of Lewis or Brønsted acids and bases, which contain various hydrogen bond acceptors (HBAs) and hydrogen bond donors (HBDs) [7,8]. DESs are considered a new class of IL analogs because they share many physical characteristics and properties but are two different types of solvents. The structure and properties of ILs and DESs are different. Ionic interactions dominate the behavior of ILs, and DESs, as a eutectic mixture of two or more different components, and they exhibit strong hydrogen bonding [9,10]. With many suitable starting materials and their possible combinations, they can modify the properties of these substances for specific applications. Therefore, amorphous complexes can be used widely in science, research, and technology.

Indomethacin (IDM), which is a type of nonsteroidal anti-inflammatory drug (NSAID), exhibits anti-inflammatory and analgesic effects by suppressing the conversion of arachidonic acid to prostaglandins by inhibiting cyclooxygenase and suppressing prostaglandin synthesis. By inhibiting sodium channels in the nerve membrane, action potential conduction is reversibly suppressed, and sensory nerves and motor nerves are blocked [11]. Flurbiprofen (FLU) is also an NSAID used to treat the signs and symptoms of osteoarthritis and rheumatoid arthritis. FLU may also be used topically before ocular surgery to prevent or reduce intraoperative miosis. FLU is structurally and pharmacologically related to fenoprofen, ibuprofen, and ketoprofen [12]. With these NSAIDs, some researchers have used drug combinations to develop co-crystal systems to facilitate transdermal drug delivery [13]. Among them, local anesthetics are often used.

**Figure 1:**
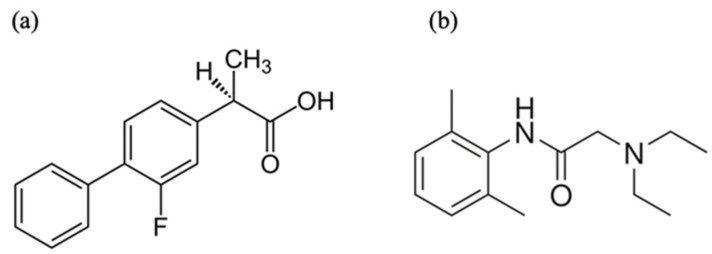
Chemical structures of the investigated molecules (**a**) Flurbiprofen (FLU) and (**b**) lidocaine (LDC).

Lidocaine (LDC) is a local anesthetic and has applications in several superficial and invasive treatments. Since its discovery and availability for sale and use in the late 1940s, LDC has become a very commonly used medication [14]. Among the various routes of clinical use, some researchers have focused on the complex formed by NSAIDs and LDC because it can improve the bioavailability of the drug through routes such as the transdermal route [15]. DES has similar physical components and properties as IL but possesses different chemical properties. The chemical structures of FLU and LDC are shown in Figure 1.

From the previous report, we obtained a reconstituted viscous paste from IDM, and LDC, which is a local anesthetic ester in the form of liquids, creams, and gels used to relieve pain and discomfort. When kneaded with an appropriate amount of water, it was revealed that this paste is a molecular complex [16]. The skin permeation of IDM from the novel IDM/LCD complex was greater than IDM alone [17]. Until now, LDC has been proven to form a complex, or ILs with other NSAIDs. There have been combinations with ibuprofen to form DES [18], naproxen to form IL [19], diclofenac to form IL [20], aceclofenac to form DES [21], and etodolac to form IL [22] reported.

A transdermal drug delivery system (TDDS) can be used, alternatively, to minimize and avoid the limitations of the oral and parenteral administration of drugs [23]. Presently, various drugs have been applied to the TDDS and approved by the US Food and Drug Administration (FDA), such as nicotine patches, estradiol, fentanyl, and rotigotine [24,25,26]. However, the skin, as the largest tissue in the human body, is a barrier that limits the degree and speed of drug absorption by TDDS [27]. To overcome these problems, ILs and DESs have emerged as medications with potential use as facilitators of dermal absorption [28,29]. ILs and DESs can increase skin permeability by increasing the partition coefficient of the administered drug or its thermodynamic action and momentarily modifying the composition of the stratum corneum by breaking down the lipid structure [30,31,32]. Other enhancement mechanisms, such as decreased melting point and increased membrane solubility, are believed to be related to the permeation-enhancing capability of coformers such as menthol [33,34]. Indeed, the oily liquid phase (active pharmaceutical ingredient DES) containing a high drug concentration acts as a reservoir and thus may provide a higher driving force for the diffusion of drug molecules into the skin [35]. Some NSAIDs and LDC are used as external preparations for analgesia and anti-inflammatory purposes. The complex formed by NSAIDs and LDC may change the physical and chemical properties, thereby improving skin penetration efficiency [36,37,38]. Therefore, it is necessary to investigate the effect of the prepared complexes on the transdermal ability of NSAIDs.

Therefore, we worked on forming an amorphous molecular complex in this study by combining various NSAIDs (FLU, etodolac, IDM, loxoprofen sodium, naproxen, and piroxicam) and LDC, and the physicochemical properties of the newly prepared complex were examined. The focus was on the intermolecular interaction between an NSAID (FLU) and LDC, and the formation of a complex was confirmed using attenuated total reflection (ATR)-Fourier transform infrared spectroscopy (FTIR), thermal analysis, ^13^C dipolar decoupling (^13^C DD), ^15^N cross-polarization (^15^N CP), magic-angle spinning (MAS), and nuclear magnetic resonance (NMR) measurements. Hypromellose (HPMC) hydrogel and white petrolatum as the base were selected to investigate the skin permeability of the obtained complex. Finally, a novel transdermal system containing a complex of FLU and LDC for alleviating chronic pain and an inflammatory disease was developed.

## 2. Materials and Methods

### 2.1. Materials

FLU was purchased from Tokyo Kasei Kogyo Co., Ltd. (Tokyo, Japan). Etodolac, IDM, loxoprofen sodium, naproxen, piroxicam, LDC (for biochemistry), and ethanol (special grade) were purchased from Wako Pure Chemical Industries, Ltd. (Osaka, Japan). As the base for the skin permeability test, a gel base prepared by hydroxypropyl methylcellulose (HPMC, ~15 mPa·S, 2% in H_2_O (25 °C), Sigma-Aldrich Japan K.K., Tokyo, Japan) to 2 *w/w*% and white petrolatum (JP grade, Yoshida Pharmaceutical Co., Ltd., Tokyo, Japan) were used. A Franz-type diffusion cell (Osawa Shokai Co. Ltd., Tokyo, Japan) was used. Phosphate buffer solution (PBS, pH 7.4) was prepared from one tablet of phosphate buffered saline (PBS) (Sigma-Aldrich Japan, Tokyo, Japan) dissolved in 200 mL water. All other reagents used were commercially available special-grade products.

### 2.2. Preparation of NSAIDs/LDC Complexes

NSAIDs and LDC were weighed as 200 mg in total in a glass screwed vial. The molar ratio of NSAIDs and LDC is 1:1. The vial was then stirred using a vortex mixer for 5 min to obtain the physical mixture (PM). A small amount (about 800 μL) of ethanol was added to this PM, heated to 80 °C to dissolve, and kept at 40 °C for 12 h. Finally, it was dried under reduced pressure for 24 h, and a gel-like sample (defined as Complex) was obtained.

### 2.3. Physicochemical Properties of FLU/LDC Complex

#### 2.3.1. ATR-FTIR Measurements

The infrared spectra were determined by the ATR method using an FTIR-4200 spectrometer (Jasco Co., Tokyo, Japan) and ATR unit (ATR PRO 670H-S, Jasco, Tokyo, Japan) with an internal reflection element (a diamond trapezoid having 45° entrance and exit faces). The detector used was a mercury cadmium telluride detector (MCT-4000M, Jasco, Tokyo, Japan). The spectra were acquired from 4000 to 400 cm^−1^ with a resolution of 4 cm^−1^ at 25 °C; each sample was scanned 64 times.

#### 2.3.2. Differential Scanning Calorimetry (DSC) Measurements

DSC measurements were carried out using a Thermo plus EVO DSC 8230 (Rigaku, Tokyo, Japan) with a gas selector (Rigaku) and liquid nitrogen (LN_2_) controller. An approximately 3–5 mg sample was weighed into the aluminum pan and sealed with the aluminum lid. Al_2_O_3_ was used as a reference. The measurement temperature was −100 °C–130 °C, with a temperature rise rate of 5 °C/min.

#### 2.3.3. ^13^C Dipolar Decoupling (^13^C DD), ^15^N Cross-Polarization (^15^N CP), Magic-Angle Spinning (MAS), and Nuclear Magnetic Resonance (NMR) Measurements

^13^C DD/MAS and ^15^N CP/MAS NMR measurements were recorded on an AVANCE III 600 spectrometer (Bruker Corporation, Billerica, MA, USA) with a frequency of 150.91 MHz for ^13^C. The spectrometer was equipped with a 4.0 mm MAS probe head. The repetition delay time was 15 s; the MAS speed was 15 kHz for FLU and LDC and 5 kHz for the complex. Adamantane was used as an external standard (29.47 and 38.52 ppm, respectively).

### 2.4. Solubility Test

FLU, LDC, PM, and the Complex were added in excess to 3 mL of water. The samples were shaken at a rate of 120 times/minute for 24 h and the skin surface temperature of 32 °C. The suspension obtained after shaking was filtered through a polytetrafluoroethylene membrane filter (0.45 μm), and the filtrate was appropriately diluted to prepare a sample solution. The drug concentration in the sample solution was calculated by the high-performance liquid chromatography (HPLC) method.

### 2.5. In Vitro Skin Permeability Test

The in vitro skin permeability test was performed with minor modifications in the methods previously reported [39,40]. The Complex was weighed with the concentration of FLU and LDC (1% *w/w*) and mixed with the HPMC gel using a pencil blender and with white petrolatum using an ointment plate and spatula, respectively. The skin permeation test gel sample with a total weight of 2 g was obtained. The preparations containing pure FLU and LDC were prepared the same way as the control group.

All animal experiments were carried out in accordance with the guidelines of the Institutional Animal Care and Use Committee (School of Pharmacy and Pharmaceutical Sciences, Hoshi University, Tokyo, Japan). All procedures using animals were carried out according to protocols approved by the Animal Care and Use Committee of Hoshi University (approval number P21-076, 12 May 2021). Furthermore, all ARRIVE guidelines for the care and use of laboratory animals, the U.K. Animals (Scientific Procedures) Act, 1986 and associated guidelines, and the EU Directive 2010/63/EU for animal experiments were followed.

The excised skin of hairless mice (Labo Skin, HOS: HR-1 male, 7 weeks, Hoshino Laboratory Animals, Inc., Ibaraki, Japan) was mounted on a Franz-type diffusion cell (Vertical Diffusion Cell™, Hanson Research, Los Angeles, CA, USA) as shown in Figure S1. The donor cell was installed facing toward the stratum corneum side, and the receiver cell was forwarding the dermis side. Then, 7 mL phosphate buffer (pH 7.4), preheated to 32 °C, was added to the receiver cell. The prepared complex was applied to the donor cell (applicable area: 1.77 cm^2^), containing 2 mg drug. Then, 0.5 mL of the receiver solution was collected at predetermined time intervals for 24 h, and each aliquot was replaced with 0.5 mL of fresh PBS to keep the volume of the receptor solution constant. The drug concentration in the collected solution was measured by HPLC, and the drug skin permeation rate (flux) and cumulative permeation amount were calculated from the obtained values.

### 2.6. High-Performance Liquid Chromatography (HPLC) Measurements

The HPLC equipment of ChromNAV Chromatography Data System, with an autosampler (AS-2055plus), pump (PU-2080plus), ultraviolet-visible detector (UV-2075plus), column oven (CO-2060plus), and solvent deaerator (DG-2080-53), manufactured by Jasco Corporation (Tokyo, Japan), was used. The mobile phase comprised a phosphate buffer at pH 5.9: acetonitrile = 65:35, and the stationary phase was an Inertsil^®^ ODS-3 (4.6 × 150 mm, 5 µm, GL Sciences Co., Ltd., Tokyo, Japan). The measurement conditions were 40 °C for the column oven, 1 mL/min for the flow rate, 20 μL for the injection volume, and 254 nm for the measurement wavelength. Phosphate buffer pH 5.9 was prepared by dissolving 6.8 g of potassium dihydrogen phosphate in 800 mL of water, adding diluted potassium hydroxide (0.1 g/mL) to adjust the pH to 5.9, and then adding water to 1000 mL.

### 2.7. Data Analysis of In Vitro Skin Permeability Test

The cumulative amount of drug permeated through the skin was plotted as a function of time. Subsequently, the flux was calculated from the slope of the linear region of this plot and expressed as (μg/cm^2^/h). The lag time (h) was determined by finding the linear portion of the cumulative amount versus time plot and extrapolating back to the *x*-axis.

## 3. Results and Discussion

### 3.1. Amorphous Molecular Complex Formation Screening between NSAIDs and LDC

Amorphous molecular complexes of various NSAIDs and LDC were prepared by the method described above; the appearance of these complexes is shown in Figure S2. Comparing the appearance with each other, it was obvious that the FLU/LDC, etodolac/LDC, IDM/LDC, and naproxen/LDC complexes developed a gel-like liquid state at room temperature. However, the Lox/LDC and Pir/LDC complexes developed a hard crystalline state at room temperature. From these complexes, IDM/LDC and Pir/LDC complexes were yellow, the Lox/LDC complex was white, and other complexes were transparent. It has already been reported that the etodolac/LDC complex was an IL [22] and the IDM/LDC complex was reported to form a water-soluble complex as described previously [16]. On the other hand, the naproxen/LDC complex was judged not to be a molecular complex because no interaction with LDC was observed according to our method as a result of the preliminary test. However, Fiandaca et al. reported that naproxen and LDC form an IL material at a peritectic point corresponding to 2 mol of naproxen to 1 mol of LDC [19]. The method described in the study by Fiandaca et al. used tetrahydrofuran to prepare IL, whereas we used ethanol, which could have led to different results.

On the other hand, the interaction was confirmed from the preliminary test results regarding the gel-like mixture formed by the combination of FLU and LDC. Few reports of amorphous complex of FLU and LDC for a novel transdermal system and their interaction mechanisms have been shown. For defining it as co-amorphous, IL, or DES, detailed physicochemical properties of the gel-like compound was subsequently analyzed.

### 3.2. Physicochemical Properties of FLU/LDC Complex

To explore the physical and chemical properties of the FLU/LDC complex (Complex), we performed ATR-FTIR, DSC, and MAS/NMR measurements on the Complex and compared it with the pure drug to clarify the changes in its physical and chemical properties.

#### 3.2.1. ATR-FTIR Measurements

**Figure 2:**
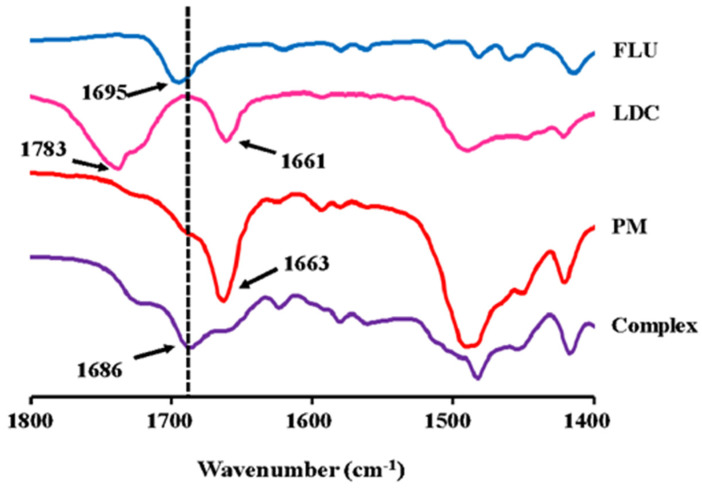
ATR-FTIR spectrum for the FLU, LDC, PM, and FLU/LDC complex.

Figure 2 shows the results of ATR-FTIR measurements for the FLU, LDC, PM, and the Complex. ATR-FTIR spectroscopy has been considered a useful tool for determining molecular complex formation. For the FLU, the stretching vibrations of the carboxylic acid carbonyl group were detected at 1695 cm^−1^. The amide carbonyl stretching vibrations for the LDC were recorded at 1661 cm^−1^. This recorded FTIR spectrum and band assignment correlate with the previous reports [12,19,41,42].

In the Complex, the peaks of 1695 cm^−1^ derived from the carboxy group observed in FLU and 1783 and 1663 cm^−1^ derived from amide I observed in LDC can be seen faintly, covered by a larger peak at 1686 cm^−1^. All these peaks disappeared in the PM sample. The Complex sample had a peak shift to 1686 cm^−1^ that differed from that in pure FLU, LDC, and PM samples. These results were consistent with the previous studies of the combination between other NSAIDs and LDC [21,22]. The change in the electron density of FLU molecules after the complex formation could explain the peak shift. These spectrum changes suggested a hydrogen bond formed between FLU and LDC in the complex sample. Because only the peak shift had occurred and no new peaks appeared, it also reflected that a hydrogen bond may have formed between LDC and FLU, and there was no clear sign of ionization or proton transfer. Recently, Agarwal et al. reported that a eutectic formation was obtained by combining LDC and myristic acid at a molar ratio of 1:1 because of a loss in the dimeric conformation of myristic acid, resulting in hydrogen bonding of its −COOH group with amide I moieties of LDC [43]. Furthermore, a frequency of 1664 cm^−1^ for the amide C=O stretching mode in pure LDC is typical for hydrogen-bonded amide carbonyls. This band decreases in intensity in the binary mixtures with increasing myristic acid. We speculate that these observations indicate that the intermolecular hydrogen bonding between the amide −C−O and −N−H of neighboring molecules within LDC is reduced or lost in the binary mixtures with myristic acid and is replaced by hydrogen bonding between −N−H of the LDC and −C=O of myristic acid and also −C=O of the LDC with myristic acid −OH. These observations suggest that the intra- and intermolecular hydrogen bonding within myristic acid alone, as well as the intermolecular hydrogen bonding within LDC alone, are each replaced by weaker intermolecular bonds formed between LDC and myristic acid in the binary mixtures, which explains the relatively lower melting point of the binary mixtures in comparison with that of LDC alone and myristic acid, respectively [43]. Consequently, it could be inferred that a relatively weak hydrogen bond is formed between the carboxyl group of FLU and the amide group of LDC.

#### 3.2.2. DSC Measurements

**Figure 3:**
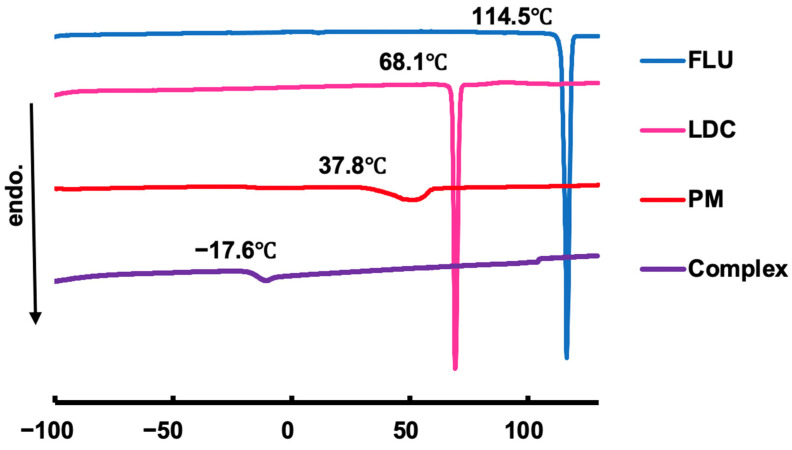
DSC profiles for the FLU, LDC, PM, and FLU/LDC complex.

Figure 3 shows the results of the DSC measurement of FLU, LDC, PM, and the Complex. The melting point was confirmed at about 115 °C for FLU and about 68 °C for LDC, which correlated with the published data [42]. In the PM sample, an endothermic peak considered a co-melting point was observed at about 38 °C. On the other hand, no clear endothermic peak was observed for the Complex sample in the measured temperature range, suggesting that the Complex had no melting transition and was in an amorphous state. In the Complex, a baseline change was observed at about −18 °C, indicating that glass transition may have occurred before and after this temperature. Combined with the FTIR results, all these changes proved that the developed system can be considered a eutectic system and that the Complex had formed an amorphous state.

#### 3.2.3. ^13^C DD and ^15^N CP/MAS NMR Measurements

**Figure 4:**
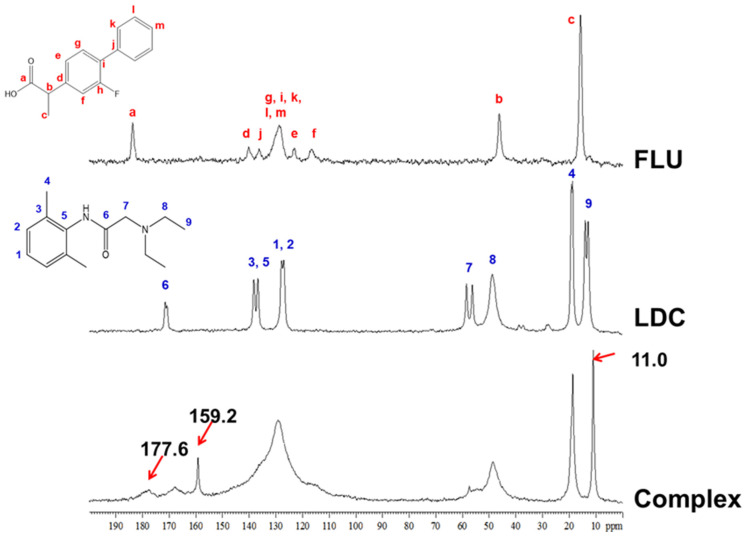
^13^C DD/MAS NMR spectra of FLU, LDC, and FLU/LDC complex.

MAS NMR enables the study of ILs and DESs over a wide range of temperatures and their characterization in liquid and solid states in a single setup. There are a very limited number of previous MAS NMR studies on ILs or DESs; these have focused on solutes dissolved in the IL or DES or interactions of the IL or DES with other materials or biological molecules rather than directly probing intermolecular interactions between the molecules of the neat ILs or DESs themselves [44,45,46]. Therefore, the ^13^C DD/MAS NMR experiment was used to investigate the interaction between FLU and LDC in the complex. The NMR spectra of FLU, LDC, and the Complex are shown in Figure 4. In the Complex, the signals at 11.0 and 159 ppm were observed, which were not observed in pure FLU and LDC. The peak derived from the carbon at position a, which forms the carboxy group of FLU, was observed at around 184 ppm in pure FLU, whereas it was shifted to about 178 ppm in the Complex. The peak derived from the aromatic ring, observed at around 110–140 ppm in the FLU and LDC alone, was broadened in the Complex.

**Figure 5:**
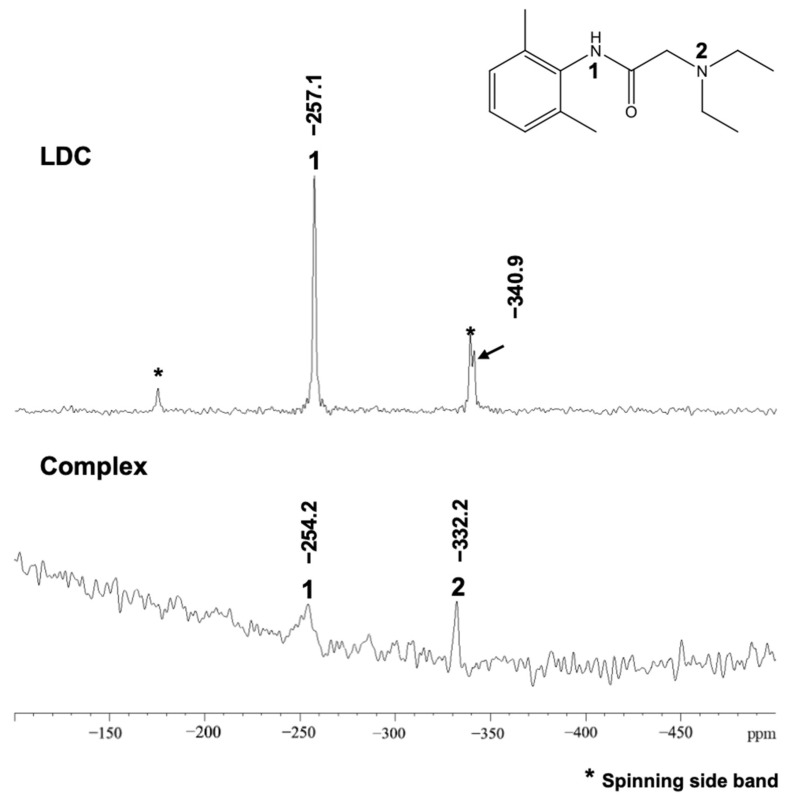
^15^N CP/MAS NMR spectra of FLU, LDC, and FLU/LDC complex.

Figure 5 shows the results of ^15^N CP/MAS NMR measurements that were performed to observe the nitrogen signals of the LDC alone and in the Complex. The results showed that the signal position of tertiary amine in the Complex was shifted to a much higher field (−340.9 ppm to −332.2 ppm) than that in LDC alone, suggesting that the carboxyl group of FLU interacted with the tertiary amine of LDC.

From these results, it was suggested that not only the ionic interaction between the carboxyl group of FLU and the secondary amine of LDC but also the Π-Π interaction between the aromatic rings of FLU and LDC contributed to the molecular complex formation. Moreover, ATR-FTIR measurements suggested that the carboxyl group of FLU and the amide group of LDC form a relatively weak hydrogen bond. Therefore, it was difficult to clearly define the complex composed of FLU and LDC as IL or DES. The complex was categorized within a boundary between DES and partially ionized ILs [47].

### 3.3. Solubility Test

**Figure 6:**
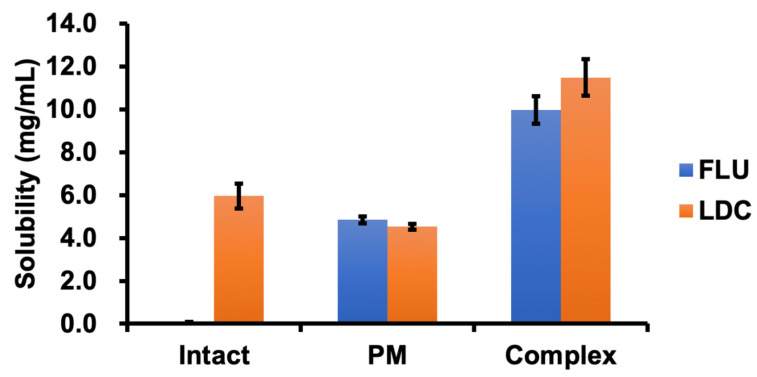
Solubility of FLU and LDC from the Complex at 32 °C (*n* = 3, the bar indicates the average ± S.D.).

Figure 6 shows a comparison of the solubility of the Complex, pure drug, and PM at 32 °C. FLU is almost insoluble in water, whereas LDC is soluble in water. The solubility of FLU in the Complex was about 10.0 mg/mL, which was about 100 times higher than that of the pure drug at 0.1 mg/mL. The solubility of FLU in PM was 4.8 mg/mL, which was about half that of the Complex. However, the solubility of LDC was 11.5 mg/mL, which was about twice as high as that of the pure drug. The solubility of LDC in PM was 4.5 mg/mL, which was about 40% of that of the Complex. Thus, the PM and the amorphous complex increased the solubility of FLU and LDC. However, the amorphous complex significantly contributed to the increased drug solubility more so than the PM.

This result was consistent with the previous report [22]. The formation of an amorphous state would reduce the melting point and thus increase the solubility in water. On the other hand, the presence of the amorphous complex in the ionized form increased the potential for the establishment of hydrogen bonds and hydration, thus allowing the drug to achieve higher solubility. Interestingly, the solubility of LDC in PM was slightly less than that of the LDC alone, but its solubility in the Complex was twice compared with that of the LDC alone. These outcomes suggest that the structure of the amorphous complex significantly increased the hydrophilicity of FLU and LDC. A comparison between the two compositions, FLU and LDC, showed that FLU exhibited significantly better solubility improvement than LDC, suggesting that the interaction between LDC and FLU in the Complex was more effective in promoting the solubility of FLU.

### 3.4. Skin Permeation Test of FLU/LDC Complex

In Japan, several transdermal formulations of FLU are commercially available for their analgesic, anti-inflammatory, and antipyretic properties. Moreover, S-flurbiprofen (S-FLU) plaster, which contains the primary active NSAID S-FLU, was launched in 2016. S-FLU is highly skin permeable and reaches a significantly higher concentration in the synovium than topical FLU [48]. Additionally, the clinical combined therapeutic effects of LID and FLU for reducing pain were enhanced [49]. Therefore, the clinical application of a transdermal formulation containing FLU and LDC would be crucial.

**Figure 7:**
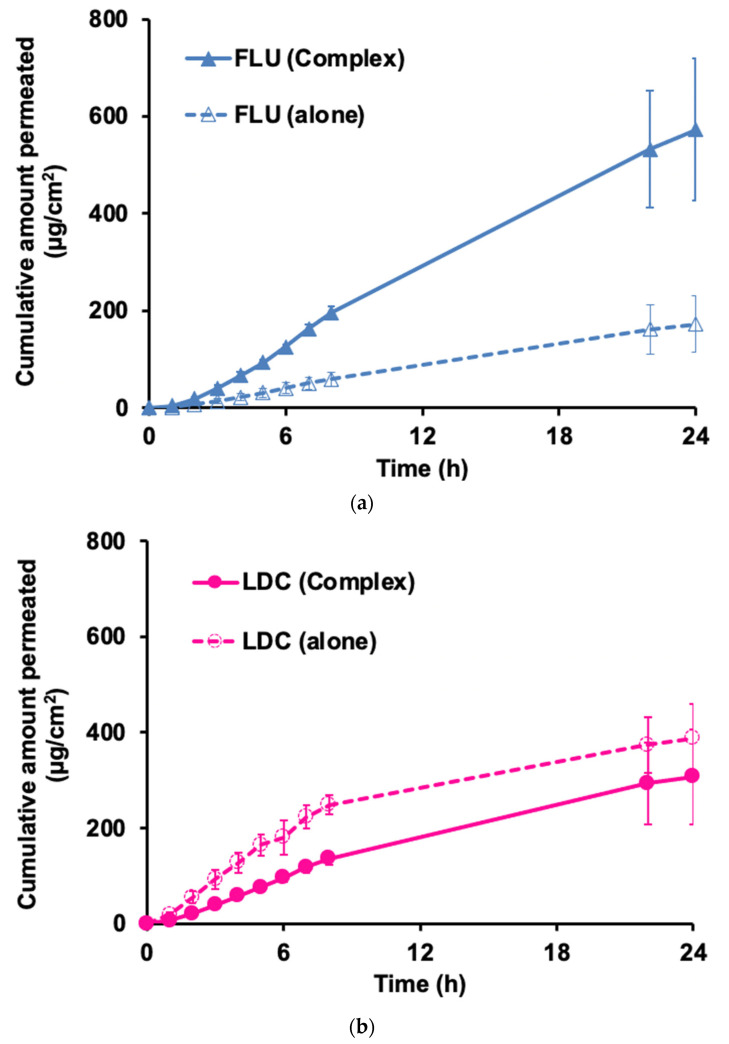

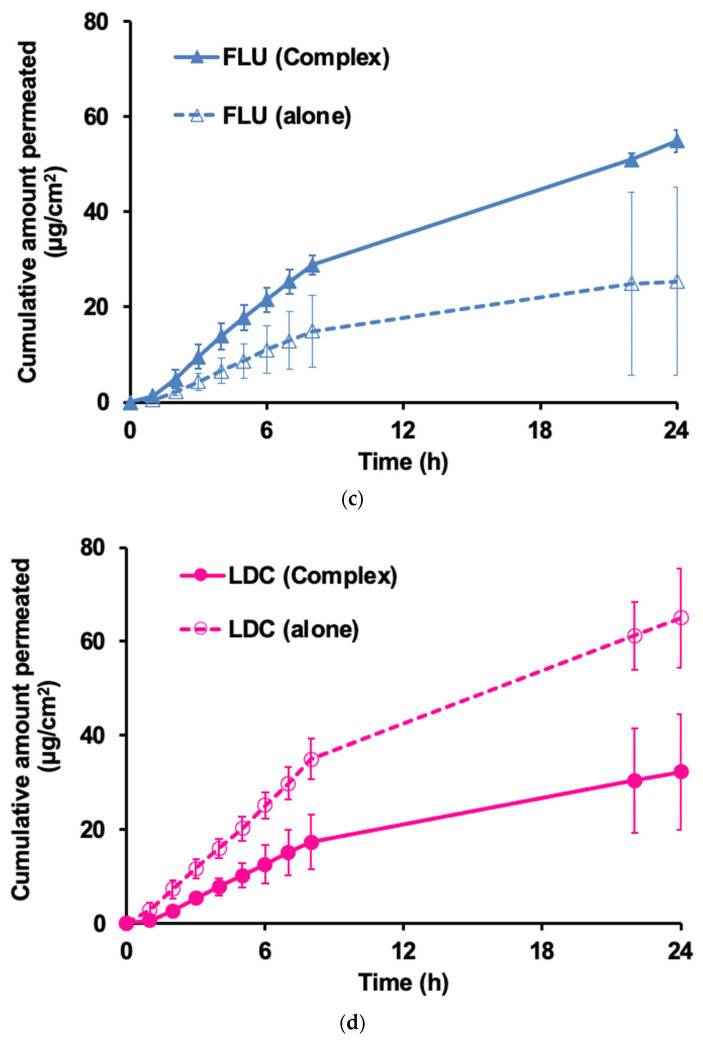
Skin permeation profile of FLU and LDC from the Complex in 2% HPMC gel (**a**,**b**) and white petrolatum (**c**,**d**).

Skin permeation amounts of FLU (Figure 7a) and LDC (Figure 7b) are shown with 2% HPMC gel used as the base. The amount of FLU permeated when white petrolatum was used as the base is shown in Figure 7c, and the amount of LDC permeated is shown in Figure 7d. The permeation parameters, flux, lag time, and cumulative amount are shown in Supplemental Tables S1 and S2 for 2% HPMC gel and white petrolatum, respectively.

Figure 7a demonstrates that when 2% HPMC gel was used as a base, the FLU alone flux was 8.0 μg/cm^2^/h, whereas that for the FLU from the Complex was 30.1 μg/cm^2^/h, with an increase of about 3.8 times (Figure 7a and Table S1). On the other hand, the flux of LDC from the Complex was about 1/2 that of LDC alone (18.4 μg/cm^2^/h) (Figure 7b and Table S1), i.e., FLU in the Complex had a better permeability than FLU alone, whereas the LDC in the Complex could not be permeated through the skin as much as from the LDC alone. From this, it was found that the combined use of LDC improved the skin permeability of FLU.

When white petrolatum was used, the flux of FLU from the complex was 4.32 μg/cm^2^/h, which was about twice the flux from the FLU alone (Figure 7c and Table S2), and for the LDC, it was similar to that with 2% HPMC gel. In the case of the Complex, the flux was 2.5 μg/cm^2^/h, about 50% lower than that of the LDC alone, 4.46 μg/cm^2^/h (Figure 7d and Table S2). It was speculated that the flux of FLU increased when using the Complex because the solubility was increased by interacting with LDC. This drug–drug interaction may also affect the structure of the stratum corneum to improve FLU permeability. On the other hand, the reason why the permeability of LDC from the Complex decreased compared with that of the pure drug is unclear, despite the fact that LDC has high skin permeability. Miwa et al. reported that when comparing the skin permeation of LDC formed as IL with etodolac (1:1, mol/mol) and LDC alone in a transdermal patch through the Yucatane Micro Pig, LDC alone showed a higher flux than LDC formed as IL [22]. They suggested that LDC–etodolac IL in the patch appeared to be able to self-sacrificially improve the limiting steps, thereby improving the skin permeation of etodolac through the matrix and stratum corneum of the skin and that both drugs might stay together in the skin as ion pairs or clusters rather than as independent solvated ions. However, the underlying mechanism behind the improved skin permeation was not clear [22]. We hypothesized that the reason for the decreased skin permeability of LDC from the Complex was consistent with their findings.

Finally, we compared the drug skin permeability of the complexes on different bases. We observed that during the initial 8 h, LDC alone exhibited better skin permeability in both bases (Figure 7b,d), followed by FLU in the Complex (Figure 7a,c). However, with time, the FLU in the Complex showed better skin permeability than the LDC alone in a 2% HPMC gel base. In both bases, the skin permeability of FLU in the Complex was greater than that of FLU alone, indicating that LDC could promote the skin permeation ability of FLU. The difference was that the cumulative amount of FLU from the Complex in the 2% HPMC gel was about six times that from FLU alone. In contrast, the cumulative amount of FLU from the Complex in the white petrolatum was only about three times that from FLU alone. When the 2% HPMC gel was used, the cumulative amount of the four samples was much greater than that with the white petrolatum base. Thus, the distribution to the skin was reduced in the white petrolatum base, and the FLU permeability was also reduced. This might be caused by the different solubilities of the drugs LDC and FLU in the two bases.

## 4. Conclusions

Because of the development of a novel transdermal system for alleviating chronic pain and inflammatory disease, we successfully obtained a gel-like mixture at room temperature by mixing FLU and LDC with ethanol from the screening of the amorphous molecular complex formation between NSAIDs and LDC. The results of ATR-FTIR, DSC, and MAS/NMR measurements proved that a molecular complex in an amorphous state was formed including a weak hydrogen bond formed between the carboxyl group of FLU and the amide group of LDC, ionic interaction between the carboxyl group of FLU and the secondary amine of LDC, and Π–Π interactions between the aromatic rings of both drugs, and we defined the Complex as IL and/or DES. Using the complex of FLU and LDC, it was found that the solubility of FLU and LDC was increased about 100 and 2 times compared with the drug alone, respectively. It was clarified that the skin permeability of FLU from the HPMC gel and white petrolatum containing the Complex was significantly improved compared with the FLU alone, whereas that of LDC from the Complex decreased by about 50% than that of LDC alone using the HPMC gel and white petrolatum, which indicated that LDC could interact with FLU to improve its ability to permeate through the skin. Therefore, developing a transdermal preparation with a novel inflammatory and analgesic effect is possible. This may prove useful for treating chronic pain related to rheumatoid arthritis, osteoarthritis, and lumbago and inflammatory diseases such as shoulder periarthritis, tenovaginitis, and cervical syndrome.

## Data Availability

Not applicable.

## Supplementary Materials

The following supporting information can be downloaded at https://www.mdpi.com/article/10.3390/pharmaceutics15020318/s1, Table S1: Flux and lag time derived from the cumulative amount and time curve of 2% HPMC gel, Table S2: Flux and lag time derived from the cumulative amount and time curve of white petrolatum, Figure S1: Diagrammatic representation of a Franz diffusion cell used in this study, Figure S2: Appearance of various NSAIDs and LDC complexes.
