# Supplementary material for: Physicochemical Properties and Transdermal Absorption of a Flurbiprofen and Lidocaine Complex in the Non-Crystalline Form
Source: Pharmaceutics. 2023 Jan 18;15(2):318. doi: 10.3390/pharmaceutics15020318 (PMC9961180; doi:10.3390/pharmaceutics15020318)
Supplement: Supplementary file 1 [file pharmaceutics-15-00318-s001.zip › pharmaceutics-2113363-supplementary.pdf]

pharmaceutics

Supplementary Materials: Physicochemical Properties and Transdermal Absorption of a Flurbiprofen and Lidocaine Complex in the Non-Crystalline Form

Qihui Xu [1,†], Takayuki Furuishi [1,*,†], Kaori Fukuzawa [1,2] and Etsuo Yonemochi [1,*]

1 Department of Physical Chemistry, School of Pharmacy and Pharmaceutical Sciences, Hoshi University, 2-4-41 Ebara, Shinagawa-ku, Tokyo 142-8501, Japan; d2081@hoshi.ac.jp (Q.X.); fukuzawa-k@phs.osaka-u.ac.jp (K.F.)
2 Graduate School of Pharmaceutical Sciences, Osaka University, 1-6 Yamadaoka, Suita 565-0871, Japan
* Correspondence: t-furuishi@hoshi.ac.jp (T.F.); e-yonemochi@hoshi.ac.jp (E.Y.)
† These authors contributed equally to this work.

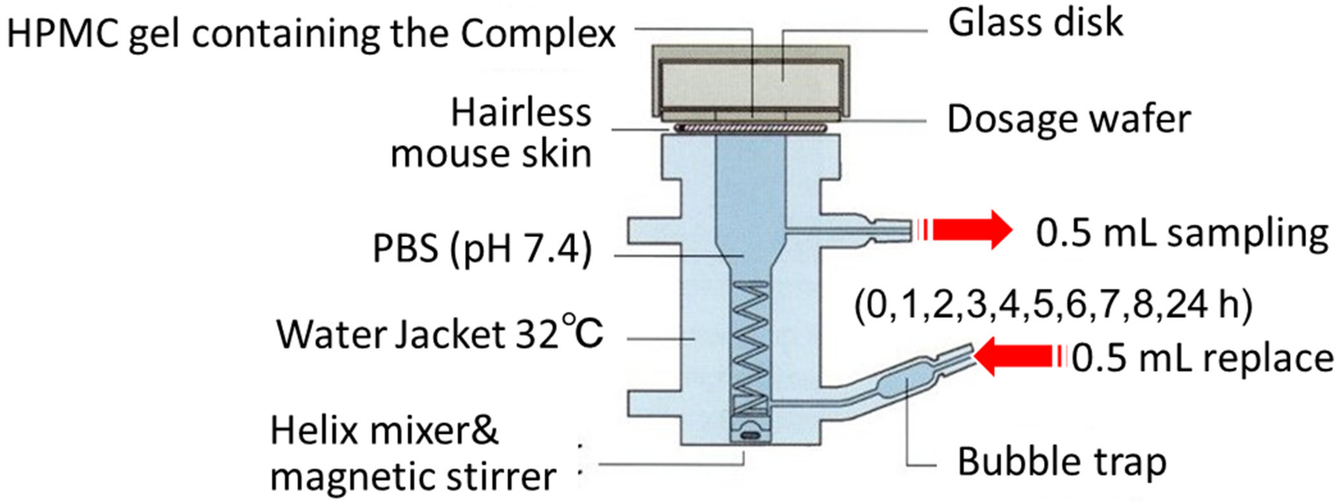

Figure S1. Diagrammatic representation of a Franz diffusion cell used in this study.

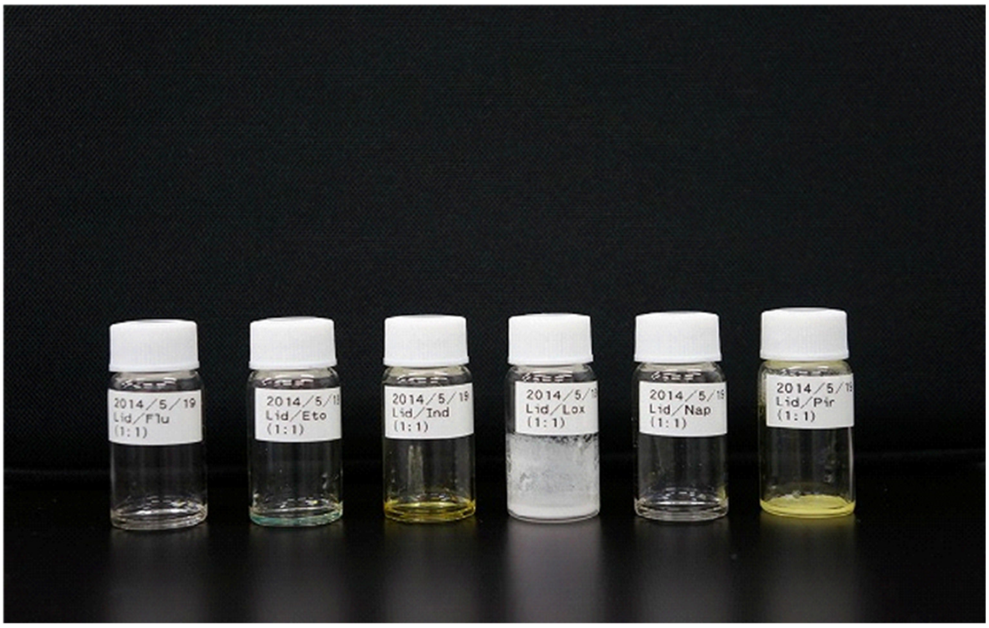

Figure S2. Appearance of various NSAIDs and LDC complex.

Table S1. Flux and Lag time derived from cumulative amount and time curve of 2% HPMC gel.

	Flux (μg/cm^2/h)	Lag time (h)	Cumulative amount permeated (μg/cm^2)
Lidocaine (LDC-FLU)	18.40±2.12	0.84±0.36	307.20±98.40
Flurbiprofen (LDC-FLU)	30.10±0.63	1.75±0.15	572.60±146.22
Lidocaine alone	36.20±2.26	0.47±0.25	387.63±71.12
Flurbiprofen alone	7.98±1.99	1.11±0.37	172.61±57.90

Table S2. Flux and Lag time derived from cumulative amount and time curve of white petrolatum.

	Flux (μg/cm^2/h)	Lag time (h)	Cumulative amount permeated (μg/cm^2)
Lidocaine (LDC-FLU)	2.49±0.58	0.87±0.19	32.24±12.21
Flurbiprofen (LDC-FLU)	4.32±0.22	0.85±0.34	54.83±2.36
Lidocaine alone	4.46±0.59	0.36±0.58	65.02±10.63
Flurbiprofen alone	2.16±0.78	0.98±0.16	25.31±19.82
